# Long Non-coding RNA RP11-395G23.3 Acts as a Competing Endogenous RNA of miR-124-3p to Regulate ROR1 in Anaplastic Thyroid Carcinoma

**DOI:** 10.3389/fgene.2021.673242

**Published:** 2021-08-05

**Authors:** An-Cheng Qin, Yi Qian, Yu-Yuan Ma, Yong Jiang, Wei-Feng Qian

**Affiliations:** ^1^The Third Affiliated Hospital of Soochow University, Changzhou, China; ^2^The Affiliated Suzhou Hospital of Nanjing Medical University, Suzhou, China

**Keywords:** anaplastic thyroid carcinoma, long non-coding RNA, microRNA, competitive endogenous RNA, bioinformatics analysis

## Abstract

Anaplastic thyroid carcinoma (ATC) is one of the most aggressive human malignancies with poor prognosis. However, the underlying mechanisms of ATC remain to be elucidated. Recently, increasing studies have focused on competitive endogenous RNA (ceRNA) to discover valuable biomarkers for the diagnosis of ATC. The present study identified 705 differentially expressed mRNAs and 47 differentially expressed lncRNAs. Gene ontology (GO) and Kyoto Encyclopedia of Genes and Genomes (KEGG) pathway analyses were also conducted. Additionally, an lncRNA/miRNA/mRNA network was constructed which included 1103 regulatory relations. The upregulation of RP11-395G23.3 in ATC cells was confirmed by quantitative reverse transcription polymerase chain reaction (qRT-PCR). In the loss of function assays, results suggested silencing of RP11-395G23.3 inhibited cell proliferation and induced cell apoptosis. Mechanically, RP11-395G23.3 could increase ROR1 via sponging miR-124-3p as a ceRNA. Moreover, ROR1 expression was decreased with the downregulation of RP11-395G23.3, but was rescued by the co-transfection of the miR-124-3p inhibitor in ATC cells. Our research suggested that the RP11-395G23.3/miR-124-3p/ROR1 axis potentially acted as a potential target for the diagnosis of ATC.

## Introduction

Thyroid carcinoma has the highest prevalence in endocrine neoplasia, and the incidence is still increasing rapidly all around the world (Pellegriti et al., 2013; Siegel et al., 2013). Normally, thyroid cancer can be classified into four types, of which anaplastic thyroid carcinoma (ATC) is found to be an uncommon but lethal malignancy with poor prognosis. Although the morbidity rate is relatively low at 1.0–2.0%, the mortality is high, taking up about 14–50% of overall thyroid cancer-related deaths (Nagaiah et al., 2011). Furthermore, conventional therapies, which include chemotherapy, radiotherapy, and surgery, have been proven to have poor effects; the survival duration is 6 months (Smallridge et al., 2009; O’neill and Shaha, 2013). Therefore, it is urgent and meaningful to investigate the underlying oncogenic mechanisms of ATC to discover valuable biomarkers for diagnosis and prognosis.

lncRNAs are defined as transcripts of more than 200 base pairs in length. Emerging evidence has showed that lncRNAs are involved critically in proliferation, apoptosis, and migration of multiple human cancers (Ma et al., 2016; Wilkes et al., 2017). Previous studies have also demonstrated that lncRNAs played an important role in ATC progression. For instance, one study suggested that lncRNA PTCSC3 prohibits the drug resistance of ATC to doxorubicin through the STAT3/INO80 pathway (Wang X. M. et al., 2018). Another research showed that MANCR downregulation significantly inhibits cancer cell proliferation and invasion, and induces apoptosis in vitro (Huang et al., 2020). Furthermore, UCA1 promotes the proliferation of ATC and provides a meaningful clue to identifying a potential target to deal with ATC (Wang Y. et al., 2018). However, the roles of most tumorigenesis-related lncRNAs in ATC remain unknown.

miRNAs, which are conserved non-coding RNAs with the length of approximately 22 nucleotides, play a determining and crucial role in tumorigenesis and metastasis by regulating the expression of target mRNAs, including thyroid cancer. For example, miR-544 has been reported to function as a tumor suppressor that inhibits the tumorigenicity of ATC (Wang et al., 2019). Triggered by miR-483, the downregulation of Pard3 increases cell invasion in ATC (Zhang X. et al., 2019). What is more, miR-650 exerts a tumorigenic function via regulating PPP2CA phosphatase in ATC (Orlandella et al., 2019).

The competitive endogenous RNA (ceRNA) hypothesis was put forward in 2011 (Salmena et al., 2011). Many studies have demonstrated that lncRNA/miRNA/mRNA ceRNA networks exert critical functions in the initiation and progression of cancers (De Oliveira et al., 2019). So far, ceRNA networks including pancreatic adenocarcinoma (Weng et al., 2020), colorectal cancer (Yang and Kang, 2020), and lung adenocarcinoma (Tang et al., 2020), even papillary thyroid cancer (Zhao et al., 2018; Liang and Sun, 2019), have already been constructed and analyzed. But due to the rarity and difficulty in tumor tissue sampling (Kunstman et al., 2015), the ceRNA network of ATC is not fully elucidated. Therefore, it is imperative to construct an lncRNA/mRNA/miRNA ceRNA network of ATC to illuminate the molecular mechanism.

In this research, we comprehensively analyzed the data from the gene expression omnibus (GEO), and screened aberrantly expressed lncRNAs and mRNAs. Then the lncRNA/miRNA/mRNA ceRNA network was constructed. In functional assays, the RP11-395G23.3/miR-124-3p/ROR1 axis was identified to be involved in the proliferation and apoptosis of ATC. Silencing RP11-395G23.3 decreased the proliferation of ATC, but increased ATC cell apoptosis via downregulating ROR1 expression. The direct regulating interaction among RP11-395G23.3, miR-124-3p, and ROR1 was verified with the dual luciferase reporter assay. These results substantially provided convincing evidence to validate the RP11-395G23.3/miR-124-3p/ROR1 axis as a useful biomarker in ATC progression.

## Materials and Methods

### Data Collection and Processing

Gene expression dataset GSE33630 including 11 ATC samples, 49 PTC samples, and 45 normal samples was downloaded from the NCBI GEO^1^ database. The microarray dataset was based on the GPL570 (HG-U133_Plus_2) Affymetrix Human Genome U133 Plus 2.0 Array platform (Barrett et al., 2007). All the probe sequences of the HG-U133_Plus_2 microarray^2^ were compared to the Genome Reference Consortium Human Build 38 (GRCh38) which was also downloaded from GENCODE (Harrow et al., 2012)^3^ using the software seqmap (Jiang and Wong, 2008).

### Identification of Differentially Expressed mRNAs and lncRNAs

The identification of differential expression of mRNA (DEG) and lncRNA (DE-lncRNA) between ATC and normal samples was carried out by Limma package (Ritchie et al., 2015) (Version 3.10.3).^4^ | logFC| > 2 and adjusted *P* value were set as cutoff criteria.

### Gene Ontology and Pathway Enrichment Analyses of DEG

Gene ontology (GO) (Ashburner et al., 2000) and Kyoto Encyclopedia of Genes and Genomes (KEGG) (Kanehisa and Goto, 2000) pathway enrichment analyses of the DEGs were performed using DAVID (Huang Da et al., 2009),^5^ DEG count ≥ 2 and *P <* 0.05 were used as the cutoff.

### Construction of ceRNA Network

In order to construct the miRNA-mRNA network, miRWalk2.0 (Dweep and Gretz, 2015)^6^ was used to predict regulatory miRNAs of candidate DEGs. To improve the predictive accuracy, the miRanda, PITA, RNA22, RNAhybrid, and Targetscan databases were also referred to. To construct the miRNA-lncRNA regulatory network, the lnCeDB (Das et al., 2014) and starBase v2.0 (Li et al., 2014) databases were utilized to predict the targeted miRNA of DE-lncRNAs which are co-expressed with DEGs. By integrating the above two networks, a comprehensive ceRNA regulatory network was constructed. Additionally, Cytoscape software version 3.6^7^ was used for the visualization of these networks.

### Cell Culture

The human thyroid follicular cell line Nthy-ori 3-1 and human ATC cell lines (C643 and HTh-7) were purchased from Shanghai Institutes for Biological Sciences, Chinese Academy of Sciences (Shanghai, China). All cells were cultured in RPMI medium (Gibco, Thermo Fisher Scientific, Inc.) supplemented with 10% fetal bovine serum (FBS), 1% penicillin, and 1% streptomycin (Gibco, Thermo Fisher Scientific, Inc.), and incubated at 37°C with 5% CO2 in a humidified incubator.

### RNA Extraction and Real Time Quantitative Reverse Transcription Polymerase Chain Reaction

Total RNA was isolated from Nthy-ori 3-1, C643, and HTh-7 cells using TRIzol reagent (Invitrogen, Thermo Fisher Scientific, Inc.) based on the protocols. Then the cDNA was synthesized using the PrimeScript^TM^RT Master Mix for the qPCR Kit (TAKARA), according to the manufacturer’s protocol. Subsequently, the cDNA was analyzed as a template in the real-time polymerase chain reaction (PCR) using Power SYBR Green PCR Master Mix (Thermo Fisher Scientific, Inc.), and the 7900HT FAST system (Applied Biosystems, Thermo Fisher Scientific, Inc.), according to the manufacturer’s protocol. The real-time PCR reaction was as follow: (1) 50°C for 3 min; (2) pre-denaturation at 95°C for 3 min; (3) denaturation at 95°C for 10 s, annealing and extension at 60°C for 30s, 40 cycles. For detecting the expression of lncRNAs and mRNAs, GAPDH (glyceraldehyde-3-phosphate dehydrogenase) was used as the internal reference gene, while to detect the expression of miRNA, U6 served as internal control. The relative expression level was normalized according to the endogenous control using the 2^–ΔΔCt^ method (Livak and Schmittgen, 2001). The PCR primer sequences are presented in Table 1.

**TABLE 1:** Primer sequences for PCR.

**Name of primers**	**Sequence (5′–3′)**
RP11-774O3.3-hF	CACTGACCGAAATGCCACC
RP11-774O3.3-Hr	GGGAGAATGCCCAGACTTGA
RP11-96D1.11-hF	GCCCATGCTGATGTACTGCTC
RP11-96D1.11-hR	CTGCCTTTGGCTGTTCTGTGA
SLC26A4-AS1-hF	AGGAGCTGGAGCTATCCTACC
SLC26A4-AS1-hR	AAGGCAGGTGGATTACGGAAG
LL22NC03-N64E9.1-hF	TCCACATTGCTTACACCATTAGTC
LL22NC03-N64E9.1-hR	CAGGTGGATTGTGGCCATTC
RP11-395G23.3-hF	AAAGGGTCCATCTCCAAGGC
RP11-395G23.3-hR	TCCGCAGGCAACAATCACA
ROR1-hF	CTGCCCACTACCAGCCAACA
ROR1-hR	CCCATTCCACCAGGATGATTT
GAPDH-hF	TGACAACTTTGGTATCGTGGAAGG
GAPDH-hR	AGGCAGGGATGATGTTCTGGAGAG
miRNA-124-3pF	TAA GGC ACG CGG TGA ATG CC
miRNA-124-3pR	GAT TGA ATC GAG CAC CAG TTA C
U6-F	CTCGCTTCGGCAGCACAT
U6-R	AACGCTTCACGAATTTGCGT

### Cell Transfection

miR-124-3p mimic, miRNA negative control (miR-NC), and miR-124-3p inhibitor were purchased from RiboBio (RiboBio, Guangzhou, China). Small interfering RNAs (siRNAs) against RP11-395G23.3 (si-RP11-395G23.3) and negative control siRNA (siControl) were purchased from Shanghai Institutes for Biological Sciences, Chinese Academy of Sciences (Shanghai, China). The sequences were: siControl: 5′-UUCUCCGAACGUGUCACGUTT-3′; si-RP11-395G23.3: 5′-GGAGUUCUCCACAUGUAAATT-3′. They were transfected into Nthy-ori 3-1, C643, and HTh-7 cells by Lipofectamine 2000 (Invitrogen, Thermo Fisher Scientific, Inc.), according to the manufacturer’s protocols.

### Cell Proliferation

Cell proliferation was analyzed using CCK-8 (Cell Counting Kit-8, Dojindo, Japan) following the manufacturer’s protocol. Twenty-four hours after transfection, 1,000 cells were seeded in each well of the 96-well plates. At the time point of 0, 24, and 48 h after treatment, 10 μL of CCK-8 solution was added into each well and incubated for 2 h. The absorbance at 450 nm was detected by a Microplate Reader (Bio-Rad Laboratories, Inc.).

### Cell Apoptosis

Annexin V-FITC/PI kits (BD Biosciences, United States) were applied to identify cell apoptosis according to the protocol. Forty-eight hours after transfection, cells were suspended with 100 μl of 1 × Binding Buffer. Then 5 μl of FITC Annexin V and 5 μl of PI were added into the solution. After incubating for 15 min avoiding light at room temperature, 400 μl of Binding Buffer was added and measured by flow cytometry (BD FACS Calibur) within 1 h.

### Western Blot Assay

Total protein from ATC cells was extracted using RIPA buffer (Beyotime Biotechnology) on ice. Then, protein concentration was quantified by a BCA Protein Assay kit (Thermo Fisher Scientific, Inc.). Identical amounts of protein (40 μg per lane) were subjected to 10% sodium dodecyl sulfate polyacrylamide gel (SDS-PAGE) electrophoresis before being transferred onto a polyvinylidene fluoride membrane (Invitrogen, Thermo Fisher Scientific, Inc.). After that, the membranes were blocked with 5% nonfat milk at room temperature for 1 h and then incubated with the primary antibodies against Ror1 (1:1,000, Abcam) and β-actin (1:1,000, Abcam) overnight at 4°C. After incubation with horseradish peroxidase-conjugated goat anti-rabbit IgG secondary antibody (1:5,000, Abcam) for 1 h at room temperature, the enhanced chemiluminescence detection system (Thermo Fisher Scientific, Inc.) was used to detect the signal; β-actin was used as an endogenous control.

### Dual Luciferase Reporter Assay

The 3′UTR of RP11-395G23.3 and ROR1 were ligated into the PmirGLO plasmid to make wild-type (wt) versions, respectively. Then the QuickChange Site-directed Mutagenesis Kit (Agilent Technologies, Santa Clara, CA, United States) was used to make mutant-type (mut) versions from RP11-395G23.3 3′UTR-wt and ROR1 3′UTR-wt. For the dual luciferase reporter assay, ATC cells were co-transfected with the constructed plasmid and with miR-NC or miRNA-124-3p mimic using Lipofectamine 2000 (Invitrogen, Thermo Fisher Scientific, Inc.). After 48 h, the relative luciferase activity of each well was measured using the Dual Luciferase Reporter System (Pro-mega, Madison, WI, United States).

### Statistical Analysis

Experimental data were exhibited as the mean ± SD, every independent experiment was carried out at least three times. SPSS 20.0 (IBM, Corp.) was used to perform statistical analyses, and GraphPad Prism 5.0 (GraphPad Software Inc.) was exploited for visualization. Differences between groups were analyzed by unpaired Student’s *t*-test, *P <* 0.05 was considered statistically significant.

## Results

### Screening of DEGs and DE-lncRNAs

**FIGURE 1:**
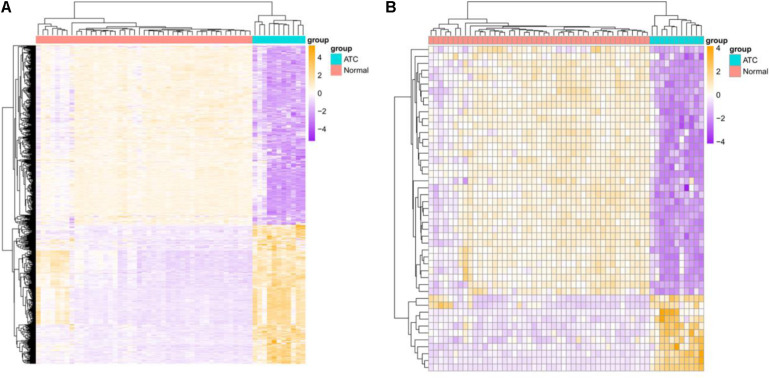
Heatmap of identified DEGs and DE-lncRNAs. **(A)** DEGs, **(B)** DE-lncRNAs. Green represents normal samples and red represents ATC samples. Up-or down-regulated DEGs and DE-lncRNAs are colored in orange or purple.

**FIGURE 2:**
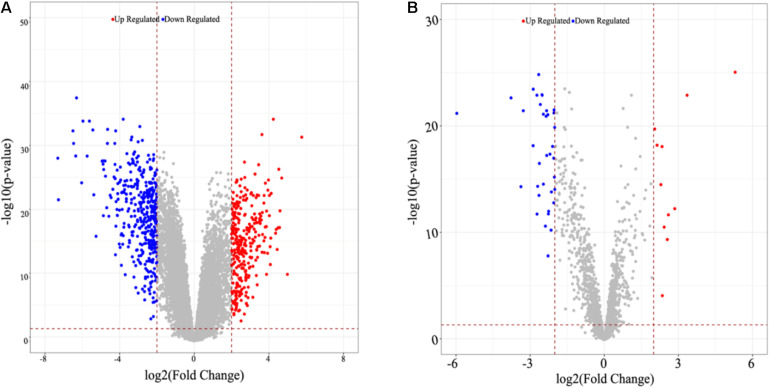
Volcano graph of identified DEGs and DE-lncRNAs. **(A)** DEGs, **(B)** DE-lncRNAs. Up-or down-regulated DEGs and DE-lncRNAs are colored in red or blue dots.

A total of 16,842 mRNAs and 3,568 lncRNAs were annotated from the sequencing data. Additionally, 705 DEGs and 47 DE-lncRNAs were screened in ATC compared to normal control, which comprised 308 upregulated and 397 downregulated DEGs, 11 upregulated and 36 downregulated DE-lncRNAs, respectively. Based on these data, a heatmap (Figures 1A,B) and Volcano graph (Figures 2A,B) were made.

### Gene Ontology and KEGG Pathway Enrichment Analysis of DEGs

**FIGURE 3:**
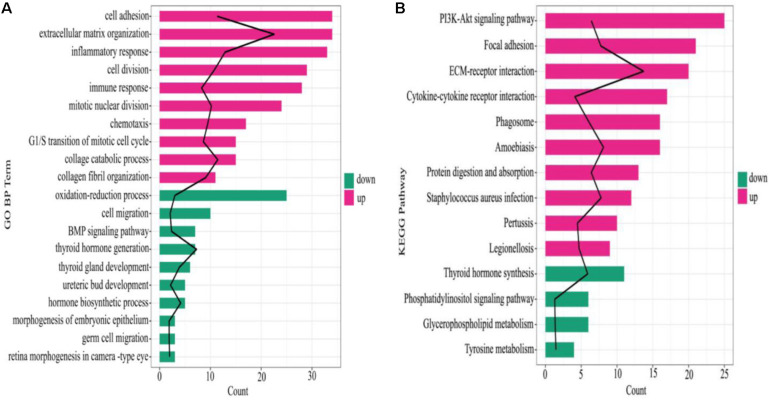
Enrichment results of GO BP and KEGG pathways. **(A)** GO BP enrichment result, **(B)** KEGG pathway. The black line denotes –log10 (*P* value), red and green color in the column represent upregulated mRNA and downregulated mRNA, respectively, the column length indicates the enriched genes number.

The GO Biological Process (BP) terms and KEGG pathways of all the DEGs were enriched, of which, the GO BP terms of the upregulated DEGs in ATC were mostly involved in cell adhesion, extracellular matrix organization, and inflammatory response, whereas the oxidation-reduction process, cell migration, and BMP (bone morphogenetic protein) signaling pathway were mostly related to the downregulated DEGs (Figure 3A). Additionally, in the KEGG pathway, P13K-Akt signaling pathway, focal adhesion, and ECM-receptor interaction included the most upregulated DEGs, while the downregulated DEGs were involved in thyroid hormone synthesis, the phosphatidylinositol signaling system, and glycerophospholipid metabolism (Figure 3B).

### Construction of ATC-Related CeRNA Network

**FIGURE 4:**
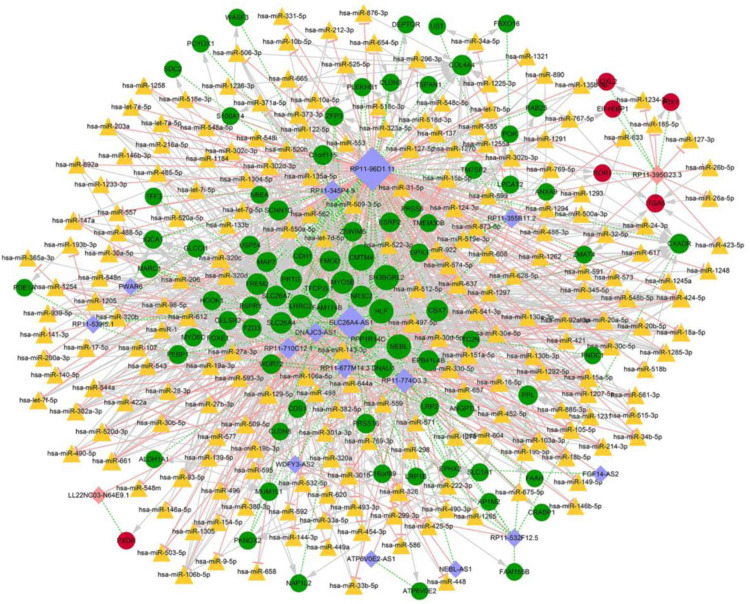
Competitive endogenous RNA regulatory network. Red circles indicate upregulated mRNAs, green circles stand for downregulated mRNAs, orange triangles represent miRNAs, pink rhombuses correspond to upregulated lncRNAs, purple rhombuses correspond to downregulated lncRNAs, light pink T shape lines suggest regulatory relationships, gray arrows suggest regulatory relationships of lncRNA-miRNA, green dotted-lines suggest co-expression relationships of mRNA-lncRNA, and size of the nods denotes the connective degree.

Overall, 4,559 miRNA-mRNA regulatory pairs were identified, including 172 mRNAs and 630 miRNAs; while 1552 lncRNA-miRNA pairs were confirmed, which contained 29 lncRNAs and 970 miRNAs. Combining the mRNAs and lncRNAs which were regulated by the same miRNA with the correlation coefficients of lncRNA and mRNA (>0.9), a total of 1,103 regulatory relations were ultimately identified, which included 215 miRNAs, 17 lncRNAs, and 89 mRNAs (Figure 4). lncRNAs RP11-774O3.3, RP11-96D1.11, SLC26A-AS1, LL22NC03-N64E9.1, and RP11-395G23.3 were identified as key regulators in the network.

### Quantitative Reverse Transcription Polymerase Chain Reaction Results of lncRNA Expression

**FIGURE 5:**
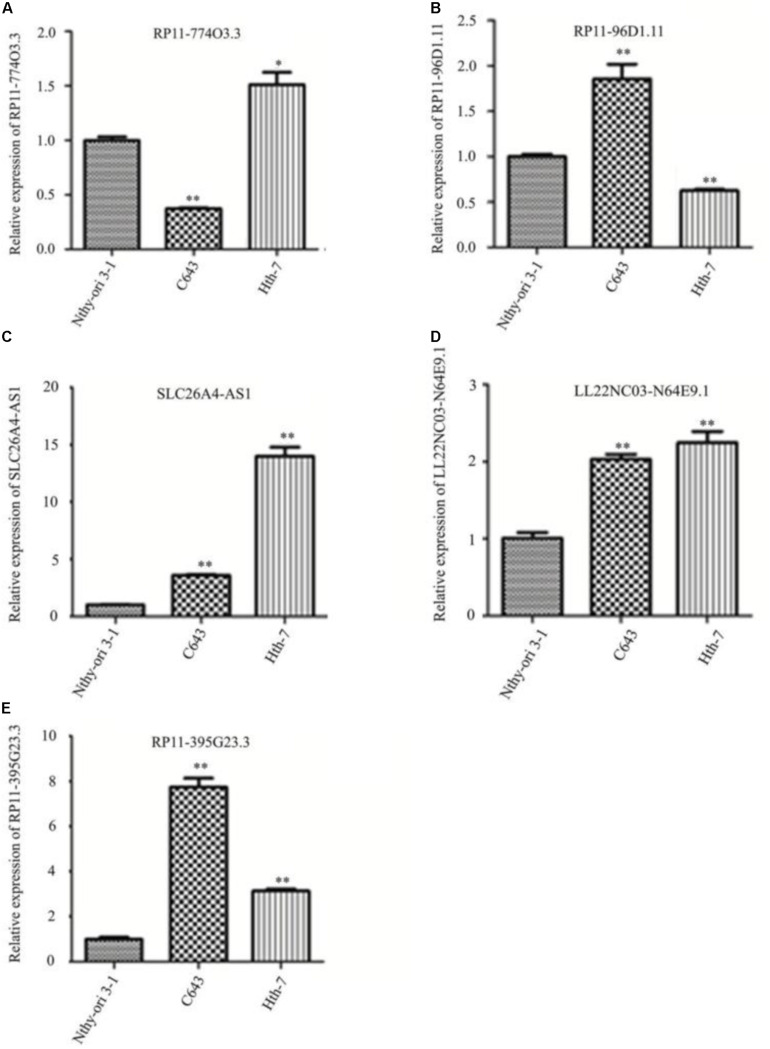
Quantitative reverse transcription polymerase chain reaction results of identified lncRNAs in different cell lines. **(A–E)** Hub lncRNAs expressions in Nthy-ori 3-1, C643, and HTh-7 cell lines. ^∗^*P* < 0.05; ^∗∗^*P* < 0.01, compared to normal cells.

Hub lncRNAs including RP11-774O3.3, RP11-96D1.11, SLC26A-AS1, LL22NC03-N64E9.1, and RP11-395G23.3 were identified according to the ceRNA network, and the verification was conducted in Nthy-ori 3-1, C643, and HTh-7 cell lines by quantitative reverse transcription polymerase chain reaction (qRT-PCR). The results showed that lncRNAs RP11-96D1.11, LL22NC03-N64E9.1, and RP11-395G23.3 were significantly upregulated in ATC cells compared to Nthy-ori 3-1, among which lncRNA RP11-395G23.3 and the corresponding RP11-395G23.3/miR-124-3p/ROR1 ceRNA network was chosen for further research (Figure 5).

### RP11-395G23.3 Enhanced Proliferation and Suppressed the Apoptosis of ATC Cells

**FIGURE 6:**
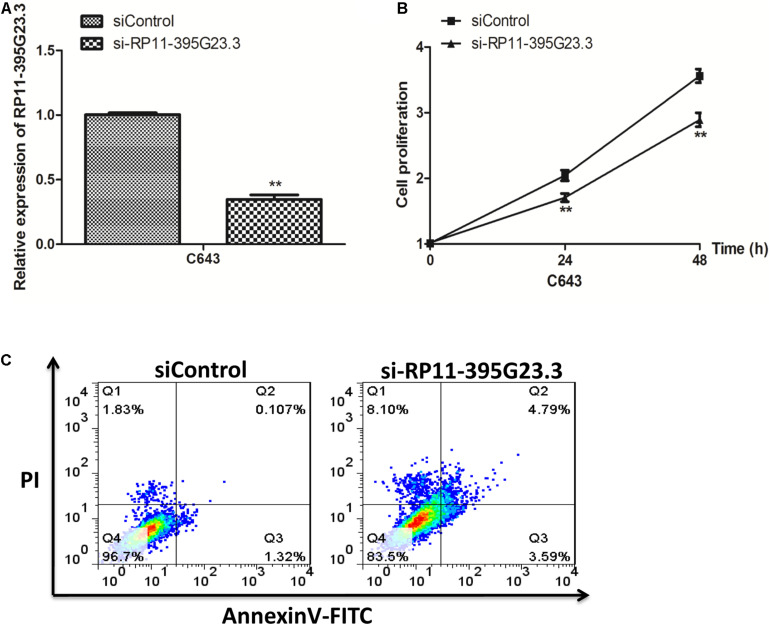
Knockdown of RP11-395G23.3 reduced cell proliferation and induced cell apoptosis in ATC. **(A)** RP11-395G23.3 expression was downregulated after the translation of si-RP11-395G23.3 in C643 cells. **(B)** Knockdown of RP11-395G23.3 reduced cell proliferation. **(C)** Silencing of RP11-395G23.3 induced ATC cell apoptosis. *P < 0.05; **P < 0.01, compared to normal cells.

To investigate the detailed function of RP11-395G23.3 in ATC, we performed loss of function assays. RP11-395G23.3 siRNA (si-RP11-395G23.3) or normal control siRNA (siControl) was transfected in C643 cells. The qRT-PCR results indicated that transfection of si-RP11-395G23.3 significantly downregulated RP11-395G23.3 expression level in C643 cells, which proved the high knockdown efficiency of si-RP11-395G23.3 (Figure 6A). After knockdown of RP11-395G23.3, a CCK-8 assay was conducted to validate cell proliferation; the results showed that the silencing of RP11-395G23.3 apparently inhibited the proliferation of C643 cells (Figure 6B). What is more, we also investigated cell apoptosis by flow cytometry, and a significantly increased number of apoptotic cells was observed in si-RP11-395G23.3-transfected C643 cells, compared to siControl (Figure 6C). These results altogether manifested that inhibition of RP11-395G23.3 could affect ATC cell biologic activity; RP11-395G23.3 played a pivotal role in proliferation and apoptosis of ATC cells.

### RP11-395G23.3 Directly Targeted and Negatively Regulated miR-124-3p

**FIGURE 7:**
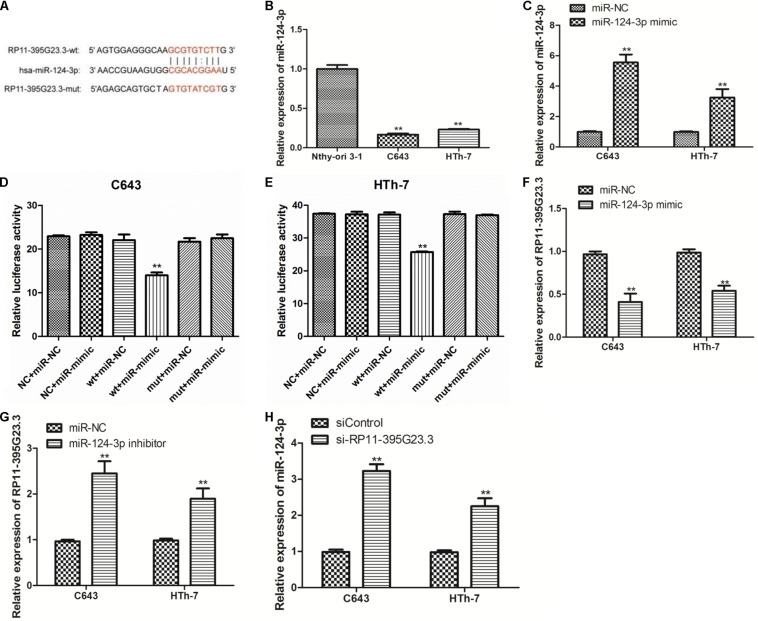
RP11-395G23.3 directly targeted and negatively regulated miR-124-3p in ATC cells. **(A)** Sequencing alignment of RP11-395G23.3 wild-type (wt) or mutant (mut) binding to miR-124-3p. **(B)** The expression of miR-124-3p was significantly downregulated in ATC cells compared to normal cell. **(C)** Transfection of the miR-124-3p mimic increased miR-124-3p expression in C643 and HTh-7 cells. **(D)** Overexpression of miR-124-3p downregulated relative luciferase activity of RP11-395G23.3-wt, but did not affect RP11-395G23.3-mut expression in C643 cells. **(E)** Overexpression of miR-124-3p reduced relative luciferase activity of RP11-395G23.3-wt but did not change RP11-395G23.3-mt expression in HTh-7 cells. **(F)** Overexpression of miR-124-3p reduced the expression of RP11-395G23.3 in C643 and HTh-7 cells. **(G)** Silencing of miR-124-3p upregulated the expression of RP11-395G23.3 in C643 and HTh-7 cells. **(H)** Knockdown of RP11-395G23.3 increased the expression of miR-124-3p in both C643 and HTh-7 cells. *P < 0.05; **P < 0.01, compared to normal cells.

Then, we further researched the underlying molecular mechanism by which RP11-395G23.3 modulates ATC cell proliferation and apoptosis. According to the prediction of lnCeDB and starBase databases, miR-124-3p harbors a potential binding site for RP11-395G23.3 (Figure 7A), and was considered to be sponged by RP11-395G23.3. To determine the hypothesis, the miR-124-3p level in Nthy-ori 3-1, C643, and HTh-7 cells was investigated by qRT-PCR, which revealed that miR-124-3p was significantly downregulated in C643 and HTh-7 cells compared to normal cells which was opposite to RP11-395G23.3 (Figure 7B), as a result, the expression of miR-124-3p was negatively correlated with RP11-395G23.3 levels. To confirm whether RP11-395G23.3 directly targeted miR-124-3p in ATC, dual luciferase reporter assays in C643 and HTh-7 cells were performed. Firstly, the efficiency of the miR-124-3p mimic was examined. The qRT-PCR results showed that miR-124-3p expression was highly upregulated when transfected with the miR-124-3p mimic (Figure 7C). Then the RP11-395G23.3-wt and RP11-395G23.3-mut reporter plasmids, along with the miR-124-3p mimic or miR-NC, were transfected into C643 and HTh-7 cells. Transfection with the miR-124-3p mimic dramatically depressed the luciferase activity of RP11-395G23.3-wt in C643 and HTh-7 cells; nevertheless, the luciferase activity of RP11-395G23.3-mut was not influenced by the upregulation of miR-124-3p (Figures 7D,E). To further confirm these findings, the miR-124-3p mimic was transfected in C643 and HTh-7 cells, and the expression level of RP11-395G23.3 was measured by qRT-PCR, the results showed that overexpression of miR-124-3p downregulated RP11-395G23.3 expression in C643 and HTh-7 cells (Figure 7F). Conversely, treatment with the miR-124-3p inhibitor upregulated RP11-395G23.3 expression in C643 and HTh-7 cells (Figure 7G). At last, to confirm the direct sponge effect of RP11-395G23.3 to miR-124-3p, si-RP11-395G23.3 was transfected in C643 and HTh-7 cells, and the expression level of miR-124-3p was increased (Figure 7H). Collectively, our results showed that RP11-395G23.3 directly targeted and negatively regulated miR-124-3p expression.

### RP11-395G23.3 Regulated ROR1 Expression via Sponging miR-124-3p in ATC Cells

**FIGURE 8:**
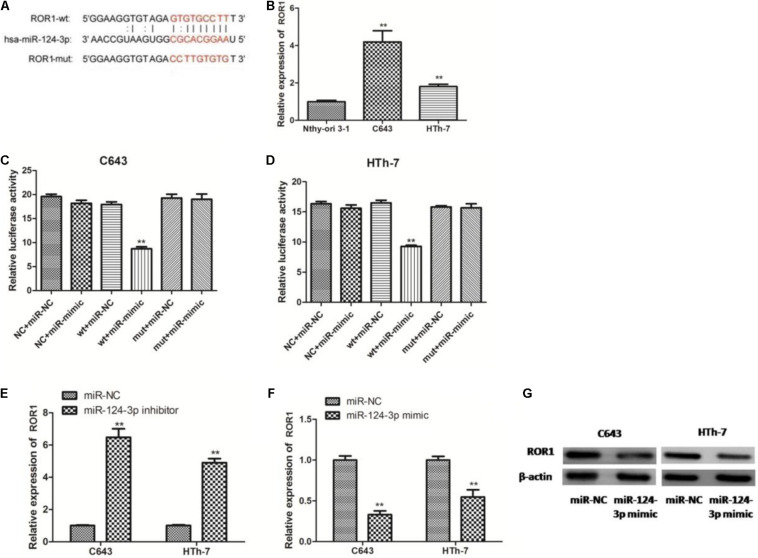
RP11-395G23.3 regulates ROR1 expression via miR-124-3p in ATC cells. **(A)** Sequencing alignment of ROR1 wild-type (wt) or mutant-type (mut) binding to miR-124-3p. **(B)** The expression of ROR1 was significantly upregulated in ATC cells compared to normal cell. **(C)** Overexpression of miR-124-3p reduced relative luciferase activity of ROR1-wt not ROR1-mut in C643 cells. **(D)** Overexpression of miR-124-3p reduced relative luciferase activity of ROR1-wt not ROR1-mut in HTh-7 cells. **(E)** Downregulation of miR-124-3p increased mRNA levels of ROR1 in both C643 and HTh-7 cells. **(F)** Overexpression of miR-124-3p decreased mRNA levels of ROR1 in both C643 and HTh-7 cells. **(G)** Western blotting showed that upregulation of miR-124-3p prohibited ROR1 protein expression in both ATC cells. *P < 0.05; **P < 0.01, compared to normal cells.

In view of these results, we tried to ascertain the possible target gene of miR-124-3p, according to the bioinformatic prediction, a large number of predominant genes were identified as potential target genes of miR-124-3p, among which ROR1 has been reported to be critically involved in tumorigenesis and inhibition of apoptosis in numerous cancers, and ROR1 harbors a potential binding site for miR-124-3p (Figure 8A). We further detected the expression level of ROR1 mRNA in normal cells and ATC cells. Compared to normal cells, ROR1 was significantly upregulated in C643 and HTh-7 cells (Figure 8B), which was negatively related with miR-124-3p. Then dual luciferase reporter assays in C643 and HTh-7 cells were performed to verify whether miR-124-3 directly targeted ROR1 in ATC cells. The ROR1-wt and ROR1-mut reporter plasmids, along with the miR-124-3p mimic or miR-NC, were transfected into C643 and HTh-7 cells. Transfection with the miR-124-3p mimic dramatically depressed the luciferase activity of ROR1-wt in C643 and HTh-7 cells; nevertheless, the luciferase activity of ROR1-mut was not influenced by the upregulation of miR-124-3p (Figures 8C,D). To further verify whether miR-124-3p downregulated ROR1, both C643 and HTh-7 cells were transfected with the miR-124-3p inhibitor or miR-124-3p mimic to decrease or increase the expression of miR-124-3p, respectively, results from qRT-PCR demonstrated that transfection with the miR-124-3p inhibitor increased the ROR1 mRNA level (Figure 8E) and transfection with the miR-124-3p mimic decreased the ROR1 mRNA level (Figure 8F). Results from western blotting demonstrated that overexpression of miR-124-3p by the miR-124-3p mimic significantly decreased the protein level of ROR1 in ATC cells compared with normal control, which was consistent with qRT-PCR results (Figure 8G). Altogether, these data indicated that ROR1 was the direct target of miR-124-3p; miR-124-3p downregulated ROR1 expression at both the mRNA level and protein level. RP11-395G23.3, miR-124-3p, and ROR1 potentially functioned in a regulatory pathway, RP11-395G23.3 acted as a ceRNA.

### miR-124-3p Reduction Antagonized the Effect of RP11-395G23.3 Downregulation on ATC Cell Viability and Apoptosis via the RP11-395G23.3/miR-124-3p/ROR1 Axis

**FIGURE 9:**
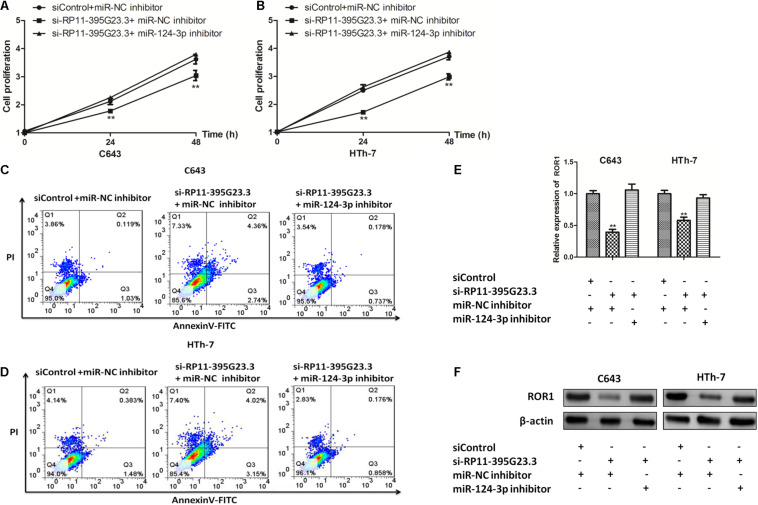
The RP11-395G23.3/miR-124-3p/ROR1 axis played a crucial role in ATC cell proliferation and apoptosis. **(A,B)** CCK-8 showed that silencing of RP11-395G23.3 inhibited cell proliferation which was reversed by the downregulation of miR-124-3p in C643 and HTh-7 cells. **(C,D)** Flow cytometry indicated that knockdown of RP11-395G23.3 induced cell apoptosis, which was attenuated by the co-transfection of the miR-124-3p inhibitor. **(E)** Depletion of RP11-395G23.3 downregulated mRNA expression of ROR1 which was ameliorated by inhibiting miR-124-3p. **(F)** Western blotting revealed that the protein level of ROR1 was decreased by RP11-395G23.3 downregulation but was rescued by restraining miR-124-3p. *P < 0.05; **P < 0.01, compared to normal cells.

To investigate the role the newly identified RP11-395G23.3/miR-124-3p/ROR1 axis played in the tumorigenesis of ATC, C643 and HTh-7 cells were co-transfected with si-RP11-395G23.3/siControl and miR-124-3p inhibitor/miR-NC inhibitor, selectively. CCK-8 was conducted to ascertain the function the ceRNA axis caused on the proliferation of ATC cells, results manifested that proliferation was inhibited profoundly in C643 and HTh-7 cells when transfected with si-RP11-395G23.3 only, but was reversed significantly when co-transfected with the miR-124-3p inhibitor (Figures 9A,B), which indicated that the miR-124-3p inhibitor could potentially attenuate the suppression induced by RP11-395G23.3. To further dig into the role the ceRNA axis played in the apoptosis of ATC cells, a flow cytometry experiment was conducted, the data showed that RP11-395G23.3 downregulation promoted the apoptosis of ATC cells, however, the effect was resisted by co-transfection of the miR-124-3p inhibitor (Figures 9C,D); RP11-395G23.3 could exert an inhibiting effect on ATC cell apoptosis through negatively regulating miR-124-3p. To validate the effect RP11-395G23.3 brings about on the ROR1 gene, loss of function and rescue experiments were carried out, the results showed that the mRNA level of ROR1 was decreased with the downregulation of RP11-395G23.3, but was rescued by the co-transfection of the miR-124-3p inhibitor in both ATC cells (Figure 9E), which specified that ROR1 was the target gene of the RP11-395G23.3/miR-124-3p interaction. In addition, the results of western blotting indicated that silencing of RP11-395G23.3 reduced ROR1 protein expression and was rescued after co-transfection of the miR-124-3p inhibitor in C643 and HTh-7 cells (Figure 9F). To sum up, the data confirmed that RP11-395G23.3 regulated ROR1 through interacting with miR-124-3p, and the RP11-395G23.3/miR-124-3p/ROR1 axis played a pivotal role in regulating the development of ATC.

## Discussion

In recent years, the incidence of ATC has increased sharply, and owing to its resistance to conventional modalities of treatment, it is necessary to define novel biomarkers and therapeutic targets. Emerging evidence has shown that the ceRNA axis plays a strong role in the pathogenesis, regulation, and prognosis of tumors. Several studies have investigated the ceRNA interaction in different types of cancers (Shen et al., 2020; Zaheed et al., 2020), but in ATC, comprehensive research of ceRNA is lacking. Due to the rarity of ATC, it is an efficient way to unveil the ceRNA axis by bioinformatics analysis and function experiments.

In this study, by analyzing dataset GSE33630 of GEO, 705 DEGs and 47 DE-lncRNAs were screened in ATC, and through bioinformatics analysis, the key lncRNAs were identified and a comprehensive lncRNA/miRNA/mRNA ceRNA network was constructed. The qRT-PCR verification of key lncRNAs showed that lncRNA RP11-395G23.3 was upregulated significantly in ATC cells compared to normal cells, suggesting that RP11-395G23.3 may exert a oncogenic role. However, little is known about the mechanism in ATC progression. The current research, for the first time, investigated the ceRNA network role that RP11-395G23.3 played in the progression of ATC, and according to the bioinformatic prediction, the RP11-395G23.3/miR-124-3p/ROR1 axis may be a potential ceRNA network.

At first, we investigated the detailed function of RP11-395G23.3 in ATC. lncRNA RP11-395G23.3, with the length of 1318 bp, is mapped to the site of 106270144-106272899 of chromosome 8, and is rarely studied, only one research indicates that RP11-395G23.3 plays a crucial role in endometrial carcinogenesis by acting as an endogenous sponge RNA to capture miR-205-5p (Xin et al., 2015), however, the relative research on other cancers is lacking. Here we showed that RP11-395G23.3 level was significantly upregulated in ATC cells compared to control cells, which suggested that RP11-395G23.3 expression is involved in the progression of ATC. In the loss of function experiment, depletion of RP11-395G23.3 significantly sequestered proliferation and induced apoptosis of ATC cells, and these results manifested that RP11-395G23.3 is an oncogenic lncRNA and could promote proliferation and inhibit apoptosis of ATC.

Then we investigated the underlying mechanism RP11-395G23.3 played in the promotion of the tumorigenesis process of ATC. Based on informatics analysis, miR-124-3p may have potential binding sites with RP11-395G23.3. In recent studies, miR-124-3p has been suggested as an inhibitor of carcinogenesis by regulating mitogen-activated protein kinase 4 in gastric cancer (Wu et al., 2020), what is more, miR-124-3p takes part in numerous ceRNA networks, such as the LINC00240-miR-124-3p-STAT3/MICA axis in cervical tumors (Zhang et al., 2020), the KCNQ1OT1-miR-124-3p-TRIM14 axis in tongue cancer (Qiao et al., 2020), the LINC01410-miR-124-3p-SMAD5 network in cholangiocarcinoma (Jiang et al., 2020), and the LINC01234-miR-124-3p-GRB2 axis in multiple myeloma (Chen et al., 2019). In our study, we manifested that miR-124-3p expression was predominantly downregulated in ATC cells, which is consistent with previous study results of low expression in papillary thyroid cancer (Sun et al., 2020), and there was an inverse correlation between RP11-395G23.3 and miR-124-3p in ATC cells. Mechanically, we also revealed that silencing of RP11-395G23.3 upregulated the expression of miR-124-3p, while overexpression of miR-124-3p prohibited RP11-395G23.3. Through dual luciferase assays, we confirmed that RP11-395G23.3 and miR-124-3p were negatively regulated by each other directly. As a result, miR-124-3p plays a pivotal regulatory function in tumors through the ceRNA network.

We further researched the target gene regulated by RP11-395G23.3 through interacting with miR-124-3p. Bioinformatics analysis predicted that ROR1 harbors potential binding sites with miR-124-3p. ROR1 (receptor tyrosine kinase-like orphan receptor 1) is an oncofetal tyrosine kinase involved in the progression of intrauterine development, which expresses highly in several neoplasms (Zhang et al., 2012). The aberrantly expressed ROR1 has been extensively suggested to play a pivotal role in the process of different varieties of cancers or malignant cells, including gastric cancer (Ikeda et al., 2020), breast cancer (Fultang et al., 2020; Stuber et al., 2020), ovarian carcinoma (Wu et al., 2019), and pancreatic cancer (Xu et al., 2018). In terms of molecular function in tumors, ROR1 has the important role of activating PI3K-AKT and MEK-ERK (Daneshmanesh et al., 2015; Zhang Q. et al., 2019), signal transducer and activator of transcription 3 (STAT3) (Li et al., 2010), cellular-mesenchymal (c-Met) (Gentile et al., 2014), epidermal growth factor receptor (EGFR) (Yamaguchi et al., 2012), and B-cell antigen receptor (BCR) (Karvonen et al., 2017). What is more, Zhang R. et al. (2019) demonstrated that the XIST/miR-30a-5p/ROR1 ceRNA network could be perceived as useful markers deciphering colorectal cancer, which was consistent with our speculation of ceRNA’s regulatory function in tumorigenesis. Our results suggested that ROR1 is an oncogenic gene and increased significantly in ATC cell lines. Additionally, the upregulation of miR-124-3p could suppress the expression of ROR1, while miR-124-3p downregulation increased ROR1 expression. The dual luciferase experiment validated that ROR1 was a direct target gene of miR-124-3p. Further experiments specified that the expression level of ROR1 was positively correlated with RP11-395G23.3. What is more, inhibiting miR-124-3p expression can attenuate the ROR1 reduction caused by RP11-395G23.3 silence. All the results demonstrated that RP11-395G23.3 functioned as the ceRNA to sponge miR-124-3p to regulate ROR1 in ATC cells.

Although it is the first study that has investigated the role of the RP11-395G23.3/miR-124-3p/ROR1 axis played in ATC, several limitations still exist. First, only one dataset of GEO was analyzed and the number of cases is limited, the result should be confirmed in additional cohorts. Secondly, due to the scarcity of ATC cases, expression verification was only carried out in ATC cells, the experiment in ATC tissues is also needed. Furthermore, ceRNA network construction was mostly based on the bioinformatics analysis and function experiments in vitro, further experiments in vivo are also required.

In conclusion, in this study we have investigated aberrantly expressed lncRNAs, miRNAs, and mRNAs in ATC samples compared to normal controls by computational analysis, and overall ceRNA networks have been constructed, which provide guidance for further research. What is more, the RP11-395G23.3/miR-124-3p/ROR1 axis is presented for the first time and can serve as potential novel candidate prognostic markers and therapeutic targets of ATC.

## Data Availability Statement

The raw data supporting the conclusions of this article will be made available by the authors, without undue reservation.

## Author Contributions

YJ and W-FQ designed the study. A-CQ and YJ collected and analyzed the data. YJ and Y-YM performed the experiments. A-CQ drafted and critically revised the manuscript. YJ critically revised the manuscript. All authors have read and approved the final version of the manuscript.

## Conflict of Interest

The authors declare that the research was conducted in the absence of any commercial or financial relationships that could be construed as a potential conflict of interest.

## Publisher’s Note

All claims expressed in this article are solely those of the authors and do not necessarily represent those of their affiliated organizations, or those of the publisher, the editors and the reviewers. Any product that may be evaluated in this article, or claim that may be made by its manufacturer, is not guaranteed or endorsed by the publisher.

## Footnotes

1
http://www.ncbi.nlm.nih.gov/geo/

2
http://www.affymetrix.com/support/technical/byproduct.affx?product=hg-u133-plus

3
https://www.gencodegenes.org/human/releases.html

4
http://www.bioconductor.org/packages/2.9/bioc/html/limma.html

5
https://david.ncifcrf.gov/

6
http://zmf.umm.uni-heidelberg.de/apps/zmf/mirwalk2/

7
http://www.cytoscape.org/
